# Retraction: Shikonin causes cell-cycle arrest and induces apoptosis by regulating the EGFR–NF-κB signalling pathway in human epidermoid carcinoma A431 cells

**DOI:** 10.1042/BSR-2015-0002_RET

**Published:** 2023-04-06

**Keywords:** apoptosis, cell cycle, epidermal growth factor receptor–nuclear factor-kappa B signalling pathway, human epidermoid carcinoma cells, shikonin

This Retraction follows an Expression of Concern relating to this article previously published by Portland Press. This article is being retracted from *Bioscience Reports* at the request of the Editor-in-Chief and the Editorial Board following receipt of a notification from a reader, alerting the Editorial Board to various images that also seem to appear in other papers by unrelated authors.

The Figure 3A flow cytometry dot plots appear similar to those found in Figure 1E from Zhang et al. 2019 (doi: 10.1186/s11658-019-0137-1) and Figure 4D from Liu et al. 2019 (doi: 10.1186/s11658-018-0130-0). The Figure 2D GAPDH western blots appear similar, once stretched and horizontally flipped, with the a-tubulin blots from Figure 2A of Zeng et al. 2015 (doi: 10.3892/ijmm.2015.2292), and the Figure 2E GAPDH bands appear similar to the B-actin bands from figure 4E of the same Zeng et al. 2015 paper. In addition, the Figure 5A B-actin bands appear similar to the GAPDH bands from Figure 6A of Wang et al 2017 (doi: 10.1590/1414-431X20165714).

The authors have been contacted with regards to the retraction and have not responded to the Journal's queries or the concerns raised. Given the extent of the issues raised, the Editorial Board stand by the decision to retract the article.

